# Intra-abdominal pectus bar migration – a rare clinical entity: case report

**DOI:** 10.1186/1749-8090-3-39

**Published:** 2008-07-03

**Authors:** Ramon Tahmassebi, Hutan Ashrafian, Caner Salih, Ranjit P Deshpande, Thanos Athanasiou, Julian E Dussek

**Affiliations:** 1 Department of Cardiothoracic Surgery, Guy's Hospital, St Thomas Street, London, SE1 9RT, UK; 2 Department Biosurgery and Surgical Technology, Imperial College London, St Mary's Hospital, London, W2 1NY, UK

## Abstract

We present the case of a 20-year-old male who underwent successful surgical correction of pectus excavatum with the Highly Modified Ravitch Repair (HMRR). At 29 months the attempted operative removal of the Ravitch bar was unsuccessful despite the impression of adequate bar location on chest x-ray. Subsequent imaging with computed tomography was unclear in determining whether the bar was supra or infra-diaphragmatic due to the tissue distortion subsequent to initial surgery. Video assisted thoracoscopic surgery (VATS) successfully retrieved the bar and revealed that it was not in the thorax, but had migrated to the intra-abdominal bare area of the liver, with no evidence of associated diaphragmatic defect or hernia. Intra-abdominal pectus bar migration is a rare clinical entity, and safe removal can be facilitated by the use of the VATS technique.

## Background

Pectus excavatum is a congenital sternal depression that occurs in approximately 8 per 1000 live births. Complications include symptoms of restrictive respiratory difficulties, mediastinal compression and body image disorders. Treatment options include conservative management for mild cases and surgery for severe symptoms or deformity [1].

Currently two surgical methods are most commonly used for correcting pectus excavatum: the Ravitch type repair and the Nuss Procedure. The most frequently applied Ravitch type procedure is the Highly Modified Ravitch Repair (HMRR), this requires an inframammary approach, resection of the deformed cartilages (perichodrectomy) followed by a sternal wedge osteotomy and placement of a retrosternal metallic bar typically stabilised by wires. The Nuss procedure avoids cartilage resection and is performed by placing a wide U-shaped bar behind the sternum through small bilateral thoracic incisions, and turning it in such a way that it protrudes the sternum and deformed costal cartilages to a desired thoracic contour [2].

Patient satisfaction can be as high as 96.5% although early postoperative complications can include pneumothorax and wound problems, whereas later complications include thoracic contour overcorrection and bar displacement [3]. We report on the late complication of pectus bar migration, which is a rare clinical entity and has only been described sporadically in the literature (Table 1).

**Table 1:** Cases of pectus bar or stabilising wire migration.

**Author**	**Procedure**	**Presentation** ** Time**	**Migration Site**	**Foreign ** **Material**	**Mechanism**	**Outcome**
Elami et el.[4] 1991	Lieberman Procedure	24 months	Right Atrium	Metallic Bar	Bar Fracture	Removed at sternotomy
McWilliams et al.[6] 1992	Ravitch Type	48 months	Right Costophrenic Angle	Stabilising Wire	Unknown	Removed at thoracotomy
Dalrymple-Hay et al.[7] 1997	Ravitch Type	9 months	Left Ventricle	Ravitch Bar	Unknown	Removed at sternotomy
Stefani et al.[8] 1998	Lodi Procedure	3 months	Peritoneum	Kirschner Wire (used as a Bar)	Unknown	Removed at Video Laparoscopy
Onursal et al.[5] 1999	Ravitch Type	48 months	Right Ventricle	Ravitch Bar	Bar Fracture	Removed at thoracotomy
Kanegaonkar et al.[9] 2001	Ravitch Type	14 months	Deep to the Sixth Rib	Ravitch Bar	Unknown	Removed at Video Thoracoscopy
Kanegaonkar et al.[9] 2001	Ravitch Type	10 months	Thoracic Cavity	Ravitch Bar	Unknown	Removed at Video Thoracoscopy
Barakat et al.[10] 2004	Morgan Procedure	24 months	Right Ventricular Epicardium	Sternal Wire	Broken Wire	Removed at sternotomy
Hoel at al.[11] 2006	Nuss Procedure	2 months	Ascending Aorta	Nuss Bar	Unknown	Removed at sternotomy
Morimoto et al.[12] 2007	Nuss Procedure	36 months	Ossified rib tissue	Nuss Bar	Unknown	Mini-thoracotomy at insertion site
Morimoto et al.[12] 2007	Nuss Procedure	36 months	Into underlying rib	Nuss Bar	Unknown	Mini-thoracotomy at insertion site
Morimoto et al.[12] 2007	Nuss Procedure	36 months	Underneath underlying rib	Nuss Bar	Unknown	Mini-thoracotomy at insertion site
Tahmassebi et al. 2008 (This case)	Ravitch Type	29 months	Bare area of Liver	Ravitch Bar	Unknown	Removed at Video Thoracoscopy

## Case Presentation

**Figure 1:**
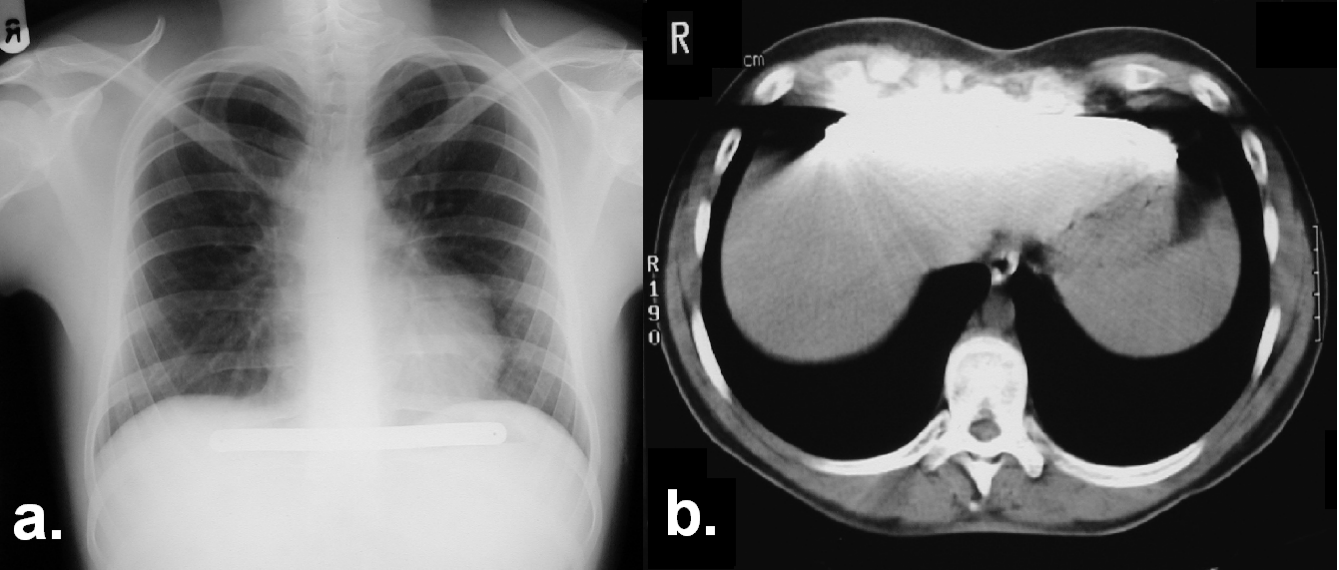
1a Preoperative chest x-ray revealing the pectus bar at the level of the diaphragm. 1b. Subsequent CT imaging of the bar in relation to the liver.

A healthy 20-year-old builder underwent surgical correction of his pectus excavatum deformity by the Highly Modified Ravitch Repair using a stainless steel pectus bar. This was placed horizontally under the sternum with its lateral extremities resting on the outer aspect of the ribs in a routine fashion. The patient was electively readmitted 29 months after surgery for removal of the bar without adverse symptoms on readmission. Pre-removal chest x-ray was unremarkable (Figure 1a).

At operative removal, the exact location of the bar could not be identified, and it was not detectable at the previous insertion site incisions. On-table X-ray image intensification simply corroborated with the original chest film that the bar was at the level of the diaphragm, but was unable to differentiate whether it was supra- or infra-diaphragmatic.

Consequently the patient underwent computed tomography (CT) in order to accurately localize the bar (Figure 1b). However, due to the nature of the corrected pectus shaped chest and the tissue distortion subsequent to surgery, its anatomical position with respect to the diaphragm was still uncertain.

A decision was made to locate the bar utilising video assisted thoracoscopic surgery (VATS). The patient underwent general anaesthesia with a double-lumen endotracheal tube, and was placed in the left lateral decubitus position with his right arm abducted. Video thoracoscopy was initiated with the introduction of the thoracoscope at the 5th intercostals space along the mid-axillary line. The other two ports were created and utilized at the 8th intercostal space along the anterior and posterior axillary lines. After sequential deflation of the lungs, thoracoscopic evaluation revealed no evidence of the bar within the chest. In conjunction with image intensification, and with guided instrument palpation the bar was located sub-diaphragmatically. To improve access, the anterior port incision was extended. A 3 cm incision was made on the diaphragm over the palpable lateral end of the bar, which was visualized, and noted to be at the superior and lateral aspect of the liver over the bare area, where it was noted to be supported by the falciform and right coronary ligaments. The bar was removed with ease, and the diaphragm was repaired using an absorbable suture. The patient had no postoperative complications.

## Conclusion

There are currently no well defined criteria to differentiate bar displacement from migration, but we propose displacement to be the movement within the boundaries of original operative tissue planes, whereas migration to occur where there is passage of foreign material through other organs, or tissues not incised during the initial operation.

Occasionally the cause of bar migration is known, when for example the sharp end of a broken metallic bar penetrates local tissues and thereby travels through them [4,5]. In other cases such as the one we report here, the cause of migration is unknown.

Possible mechanisms can include poor surgical stabilisation of operative material, trauma or local tissue erosion. This can become more likely in the presence of underlying congenital tissue defects, herniae or with raised intrathoracic pressure facilitating bar travel. The latter may have occurred in our case, as the patient was a builder and typically lifted heavy weights as part of his profession.

When bar migration does occur, its anatomical location should be confirmed by x-ray or CT. If the patient is haemodynamically uncompromised, and the bar lies within the thoracic cavity, then we advocate a minimally-invasive VATS retrieval. If however, imaging is unable to accurately discern bar location, then VATS exploration can again be beneficial, particularly as this modality can track the potential route of bar migration and retrieve it from the entry-points of destination tissues. In our case, if VATS had not been employed, laparoscopy would not have been feasible at the bare area of the liver, and an open approach via the abdomen or thorax would have become necessary.

We report this case as it is a rare complication of pectus excavatum repair, but highlights the importance of vigilant follow-up in these patients. If pectus migration does occur, then x-ray, CT and VATS may all prove beneficial in the management of such a complication.

## Competing interests

The authors declare that they have no competing interests.

## Authors' contributions

All authors contributed equally to this manuscript. All authors read and approved the final manuscript.

## Consent

Written informed consent was obtained from the patient for publication of this case report and any accompanying images. A copy of the written consent is available for review by the Editor-in-Chief of this journal.
